# Mental Health, Trauma, and Cardiovascular Risk Within South Asian Diaspora

**DOI:** 10.3390/ijerph23020250

**Published:** 2026-02-17

**Authors:** Jyoti Sinha

**Affiliations:** Sociology/Honors College, University of Massachusetts-Boston, Boston, MA 02125-3393, USA; jyoti.sinha@umb.edu

**Keywords:** trauma-informed care, public mental health, intersectionality, immigrant health, culturally responsive interventions, cardiovascular disease

## Abstract

**Highlights:**

**Public health relevance—How does this work relate to a public health issue?**

- Addresses the disproportionate cardiovascular disease (CVD) risk among South Asian immigrants as a consequence of multidimensional trauma.
- Situates physiological health outcomes within the context of systemic discrimination and immigration-related psychological stress.

**Public health significance—Why is this work of significance to public health?**

- Utilizes the “weathering” hypothesis and allostatic load framework to explain how chronic stress translates into biological cardiovascular markers.
- Highlights the critical role of community-rooted organizations (South Asian Workers’ Center-Boston) in bridging the gap between marginalized populations and formal healthcare.

**Public health implications—What are the key implications or messages for practitioners, policy makers and/or researchers in public health?**

- Advocacy for trauma-informed, culturally responsive care models that prioritize psychological safety and linguistic accessibility.
- Emphasizes the necessity of disaggregating “Asian American” health data to identify specific risks within South Asian subgroups for targeted policy intervention.

**Abstract:**

South Asian immigrants in the United States face disproportionate cardiovascular disease (CVD) risks, rooted in multidimensional trauma stemming from immigration stress, sociocultural stigma, and systemic discrimination. This paper situates these health disparities within a broader public mental health crisis, examining the intricate relationship between chronic psychological stress, intergenerational trauma, and CVD outcomes. Applying theoretical frameworks such as intersectionality and allostatic load, we explore how cumulative biopsychosocial consequences contribute to the co-morbidity of CVD and mental health disorders in South Asian communities. The study highlights SAWC-Boston’s community-based public mental health intervention, which employs culturally grounded, trauma-informed strategies to address these complex health challenges. This intervention serves as a model for addressing health disparities through community-centered approaches.

## 1. Introduction

South Asians constitute one of the fastest-growing immigrant populations in the United States, yet their health risks remain underrecognized within public health research and policy. Cardiovascular disease (CVD) represents a particularly pressing concern, as South Asian populations experience disproportionately high morbidity and mortality despite being frequently subsumed within the aggregated “Asian American” category [1,2,3]. This aggregation obscures subgroup-specific risk profiles and contributes to inadequate prevention and intervention strategies.

This process, conceptualized as allostatic load, links sustained psychological stress to cardiovascular dysregulation through neuroendocrine, metabolic, and inflammatory pathways [4,5]. This process, conceptualized as allostatic load, links sustained psychological stress to cardiovascular dysregulation through neuroendocrine, metabolic, and inflammatory pathways [4]. As a result, mental health distress and cardiovascular outcomes frequently co-occur within immigrant communities facing persistent structural adversity.

Cardiovascular disease remains the leading cause of death in the United States, accounting for more than 700,000 deaths annually [1,2]. While national surveillance systems document CVD prevalence across racial and ethnic groups, disaggregated data on working-class South Asian populations are limited. Existing evidence nonetheless indicates elevated cardiometabolic risk, including high rates of hypertension, diabetes, and premature coronary artery disease within South Asian communities [3,6,7].

Mental health stressors further compound these risks. Immigration-related strain, discrimination, and acculturative stress contribute to anxiety, depression, and trauma-related symptoms, which may exacerbate cardiovascular vulnerability by interacting with elevated cardiometabolic risk profiles and obesity-related pathways observed among South Asian Americans [8,9,10]. Barriers such as stigma, language discordance, and limited access to culturally responsive care often prevent timely intervention [9,11].

This paper situates cardiovascular disparities among South Asian immigrants within a framework of multidimensional trauma, emphasizing the intersection of structural determinants, mental health stressors, and biological risk. It further examines a community-rooted public mental health intervention model that addresses these intersecting pathways.

## 2. Background and Theoretical Framework

Understanding the health disparities faced by South Asians in the U.S. requires a framework that looks beyond individual lifestyle choices. Instead, it must acknowledge the profound influence of structural determinants of health—the conditions in which people are born, grow, live, and work. According to Solar et al. [12], these determinants, which encompass socioeconomic status, housing, and healthcare access, are the primary drivers of health outcomes.

For South Asian immigrants, these structural factors are uniquely shaped by the challenges of migration, including complex professional accreditation systems, language barriers, and discriminatory housing practices. These external pressures are often intensified by internal community dynamics, such as gender and class barriers, which further dictate how health is experienced and managed.

This structural environment directly impacts public mental health, necessitating what the Substance Abuse and Mental Health Services Administration identifies as a trauma-informed approach [13]. The relevance of this approach is supported by prior scholarship demonstrating that collective trauma shapes population-level health disparities [14]. This concept is particularly salient for the South Asian community, where the immigrant experience may involve layers of historical and cultural trauma.

The intersection of these factors is evident in recent clinical data. Evidence indicates that approximately 27% of South Asian Americans report symptoms of anxiety or depression, with significantly higher rates among individuals who have experienced discrimination [15]. When viewed through these lens, cardiovascular health disparities are not isolated physiological issues; rather, they are part of a broader public health crisis rooted in the convergence of systemic inequities and psychological trauma.

Gender, in particular, intersects with cultural norms to create unique vulnerabilities. Women may face restricted mobility, limited access to education and employment, and heightened exposure to domestic violence, all of which contribute to chronic stress and poor health outcomes. Inequalities in capabilities—including access to healthcare and material resources—disproportionately affect women [16]. In many South Asian households, women are expected to prioritize familial needs over their own health, leading to delayed or forgone medical care. Class disparities further compound these vulnerabilities. Lower socioeconomic status is associated with increased exposure to environmental stressors, limited access to healthy food options, and reduced access to quality healthcare.

To fully grasp the complexities of trauma within the South Asian community, intersectionality theory offers a powerful analytical lens. This framework posits that social identities such as race, ethnicity, gender, and class are not independent but rather interconnected, producing unique experiences of privilege and oppression [17]. For instance, a low-income South Asian woman may experience discrimination based simultaneously on ethnicity, gender, and socioeconomic status, resulting in a cumulative burden of stress. This intersectional perspective allows for recognition of the diversity of lived experiences within South Asian populations ensuring interventions account for intersectionality, such as the diverse linguistic, religious, and class-based identities within the South Asian diaspora, rather than treating it as a monolithic group.

**Figure 1:**
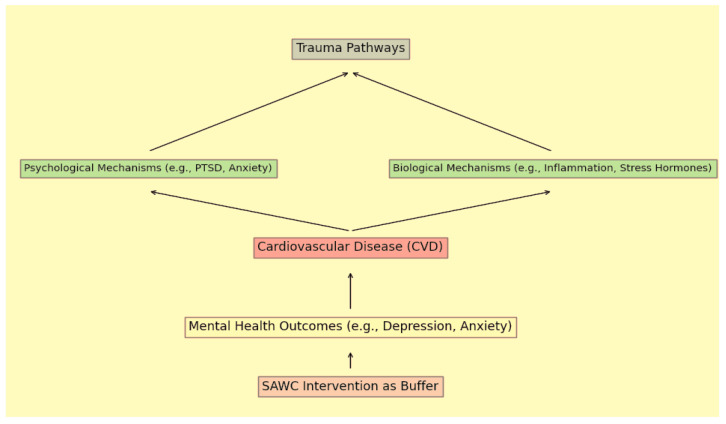
Conceptual Model of Trauma Pathways and Biological Mechanisms Leading to CVD Outcomes, with SAWC Intervention as a Protective Buffer.

The cumulative impact of these structural determinants, gender and class barriers, and intersectional stressors manifests in significant cardiovascular and mental health outcomes (see Figure 1). Extensive research demonstrates a strong association between chronic stress arising from socioeconomic disadvantage and discrimination and increased cardiovascular disease risk [4]. The constant vigilance required to navigate systemic discrimination contributes to chronic activation of stress-response systems, resulting in hypertension, inflammation, and other cardiovascular risk factors [4,5]. Moreover, mental health stigma combined with cultural expectations of resilience often prevents South Asians from seeking care for psychological distress [9]. Elevated prevalence of type 2 diabetes among South Asians further illustrates the intersection of genetic vulnerability and chronic stress exposure linked to migration and discrimination, which may also promote maladaptive lifestyle behaviors [7]. Together, these factors create a complex web of vulnerability that underscores the need for culturally tailored interventions addressing the root causes of health disparities (see Figure 1).

SAWC-Boston’s work to reduce cardiovascular health disparities among South Asian communities offers a model of community-rooted public mental health intervention that is both innovative and urgently needed. Rather than treating cardiovascular disease as a purely biomedical condition, this approach reframes it through a trauma-informed and culturally responsive lens that foregrounds the lived experiences of immigrant and working-class South Asians. This framework aligns closely with trauma-informed care principles—safety, trustworthiness, peer support, collaboration, empowerment, and cultural responsiveness—as articulated by federal public mental health guidance [13].

At the heart of SAWC-Boston’s model is the creation of psychological safety. Many South Asian immigrants arrive carrying layers of unaddressed trauma, including migration stress, discrimination, workplace precarity, family separation, and the cumulative strain of navigating unfamiliar healthcare systems. SAWC-Boston cultivates safety by offering facilitated spaces where community members can discuss health concerns without fear of stigma, shame, or medical dismissal. These spaces counter the silence surrounding mental health that persists in many South Asian households, where illness is often privatized and emotional suffering framed as personal weakness rather than a public health concern.

Trust-building is equally critical. SAWC operationalizes this principle through cultural specificity and representation, centering multilingual outreach, South Asian peer educators, and culturally knowledgeable staff. This approach counters a long history of exclusion from mainstream health research and healthcare institutions [11]. In communities where mistrust is shaped by experiences of racism, immigration surveillance, and gendered power hierarchies, such strategies are essential for encouraging engagement in preventive care and research participation. SAWC’s partnerships with Mass General Brigham and the Broad Institute reflect a collaborative model in which institutions must actively earn community trust rather than presume it.

The urgency of this work is underscored by persistent gaps in cardiovascular research. Despite South Asians comprising nearly one-quarter of the global population and experiencing disproportionately high cardiovascular disease risk—including an estimated twofold increase compared with European populations—their experiences remain underrepresented in U.S. health studies [3]. This underrepresentation is especially pronounced among working-class, low-income, and newly arrived immigrants, whose lived realities differ significantly from dominant professionalized narratives of South Asian success. SAWC-Boston’s engagement with the Our Health Study expands participation across Bangladeshi, Pakistani, Nepali, Sri Lankan, Bhutanese, and Indian communities, broadening the scope of South Asian health research.

SAWC-Boston also advances a culturally grounded public mental health model that understands culture as both a source of resilience and a site of constraint. Cultural practices such as meditation, herbal remedies, spiritual rituals, and communal eating may foster stress reduction and social belonging. At the same time, culture can reinforce hierarchical gender norms, including the “second shift,” whereby women shoulder disproportionate domestic and caregiving responsibilities alongside paid labor. These dual burdens contribute to exhaustion, shame, and social isolation, limiting help-seeking and research participation. Trauma-informed practice explicitly recognizes these gendered vulnerabilities and reframes stress as structurally, rather than individually, produced.

This trauma-informed orientation reframes cardiovascular disparities as the outcome of multidimensional trauma—structural, cultural, and emotional. South Asians often navigate hostile labor markets, exploitative working conditions, immigration precarity, and linguistic exclusion, each compounding chronic stress and biological dysregulation. In this context, SAWC’s programs extend beyond health education to intervene directly in the psychological consequences of systemic inequity. Workshops facilitated by the South Asian Workers Center create spaces where mental and physical health are understood as mutually reinforcing and shaped by working conditions, family responsibilities, socioeconomic pressures, and broader social determinants of health.

Ultimately, SAWC-Boston’s model demonstrates that improving cardiovascular health within South Asian communities requires far more than clinical intervention. It demands a public mental health strategy that addresses trauma, amplifies community expertise, and confronts the structural conditions shaping health inequities. By integrating trauma-informed principles with culturally grounded practices, SAWC-Boston offers a powerful blueprint for community-led health justice.

To combat cardiovascular disease, SAWC-Boston focuses on the following:

- Participating in healthcare studies to ensure diverse research representation;
- Providing healthcare education that empowers informed lifestyle decision-making;
- Increasing access to healthcare through navigation support and system literacy.

By addressing social, cultural, and systemic drivers of health disparities, SAWC-Boston works toward a healthier and more equitable future for South Asian communities, grounded in cultural sensitivity, trauma-informed practice, and a public mental health framework.

### 2.1. Challenges and Barriers: Trauma Responses and Research Participation

The Our Health study, despite its public health importance, faces substantial recruitment challenges within working-class South Asian communities (see Figure 1). These challenges are not solely logistical but are deeply intertwined with trauma responses shaped by historical marginalization and institutional exclusion.

### 2.2. Avoidance and Distrust

Limited digital literacy and demanding work schedules contribute to avoidance of digital research platforms, reflecting feelings of inadequacy and time scarcity [18]. Historical marginalization and perceived identity-based threats further foster distrust toward research initiatives, manifesting as protective avoidance [19,20]. Experiences of being treated as a “data bank” or “guinea pig” are closely linked to historical trauma and subsequent avoidance of healthcare systems [14]. Additionally, prioritization of immediate survival needs over long-term health reflects common responses to chronic distress and anxiety associated with elevated allostatic load [4,18].

### 2.3. Anxiety and Distress

Anxiety surrounding survey participation often stems from fears of misrepresentation and biased data use, shaped by prior experiences of stereotyping [21]. Distrust in a perceived dehumanizing and profit-driven healthcare system generates additional distress rooted in experiences of neglect and implicit bias [22,23]. Such dehumanization constitutes a traumatic experience in itself, reinforcing avoidance of healthcare engagement.

### 2.4. Trauma-Informed Approach

Addressing these barriers through a trauma-informed lens requires community-based outreach, transparent communication regarding data use, and flexible participation options. Enhancing accessibility through in-person assistance, alternative survey formats, and supportive services such as childcare is essential. Culturally appropriate communication, acknowledgment of historical injustices, and validation of community experiences are critical to fostering trust and inclusion. By recognizing trauma responses that shape research participation, public health initiatives can promote more equitable engagement and generate more accurate, impactful health evidence.

## 3. Discussion and Implications

This review underscores that cardiovascular disease (CVD) risk among South Asian immigrants cannot be adequately explained through individual behavioral or genetic frameworks alone. Instead, CVD emerges as a downstream manifestation of prolonged exposure to structural stressors and unresolved psychological trauma. Chronic stressors such as immigration precarity, racial discrimination, exploitative labor conditions, and socioeconomic marginalization contribute to sustained activation of stress-response systems, resulting in biological dysregulation across neuroendocrine, metabolic, and inflammatory pathways [3,4,17,19]. These processes increase vulnerability to hypertension, diabetes, and premature cardiovascular disease, illustrating how mental health and cardiovascular outcomes are mutually reinforcing rather than independent health concerns [6,9].

Situating these findings within a trauma-informed public mental health framework highlights the importance of understanding stress as a population-level phenomenon rather than an individual pathology [12,13]. Mental health stigma within many South Asian communities, combined with cultural norms emphasizing endurance and self-reliance, often suppresses help-seeking behaviors, allowing psychological distress to remain unaddressed until it manifests as somatic illness [7,8,14]. This dynamic reinforces a cycle in which trauma-related stress contributes to both mental health disorders and cardiovascular disease, while barriers to care prevent early intervention [9,20].

An intersectional lens further clarifies how cardiovascular and mental health risks are unevenly distributed within South Asian populations. Gender, class, immigration status, language proficiency, and employment conditions intersect to shape differential exposure to stress and access to health-protective resources [16,17]. South Asian women, particularly those in working-class households, experience compounded vulnerabilities related to caregiving burdens, restricted autonomy, and delayed healthcare utilization [15,16]. Similarly, newly arrived immigrants and individuals in low-wage or precarious employment face heightened exposure to workplace exploitation and institutional exclusion, intensifying cumulative stress and biological risk [18,19]. Recognizing this heterogeneity is essential for avoiding monolithic representations of South Asian health and for designing interventions responsive to subgroup-specific cardiometabolic risk patterns, including obesity-related cardiovascular vulnerability [10].

This analysis also reveals that barriers to healthcare and research participation are themselves expressions of trauma responses shaped by historical and contemporary marginalization. Avoidance, anxiety, and distrust toward healthcare and research institutions reflect adaptive responses to experiences of surveillance, dehumanization, and exclusion rather than apathy or disengagement [14,20,21]. Fear of misrepresentation, biased data use, and institutional harm discourages participation in public health research, particularly among working-class, undocumented, and limited English-proficient individuals [11,22,23]. Addressing these barriers therefore requires trauma-informed research practices that prioritize transparency, relational trust, and community partnership rather than solely logistical recruitment strategies [12,13].

From a public mental health systems perspective, these findings point to the necessity of culturally grounded, trauma-informed models that integrate mental and cardiovascular health promotion. Provider training must move beyond surface-level cultural competence toward institutional accountability for addressing power asymmetries, implicit bias, and linguistic exclusion within healthcare encounters [10,11,22,24]. Disaggregated data collection is equally critical for identifying subgroup-specific cardiovascular risk patterns and informing equitable policy responses for South Asian populations [3,9]. Without such data, the most vulnerable South Asian subgroups remain invisible within national prevention and surveillance strategies [1,2].

The work of SAWC-Boston illustrates how community-rooted public mental health interventions can effectively address these intersecting challenges. By embedding principles of safety, trust, empowerment, and cultural responsiveness into health education, research engagement, and healthcare navigation, SAWC-Boston reframes cardiovascular disease prevention as a collective, justice-oriented endeavor [12,13]. This model demonstrates that improving cardiovascular outcomes requires addressing the psychological consequences of structural inequity while amplifying community expertise as a central public health asset [14,18].

## 4. Conclusions

This manuscript demonstrates that cardiovascular disease disparities among South Asian communities in the United States are deeply embedded in a broader public mental health context shaped by structural inequities and cumulative trauma. Chronic exposure to immigration-related stress, discrimination, gendered labor expectations, and socioeconomic marginalization functions as an upstream driver of both psychological distress and cardiovascular risk. Understanding these conditions as interconnected rather than separate health challenges is essential for advancing equitable prevention and care.

By foregrounding trauma as a population-level phenomenon and applying an intersectional framework, this analysis challenges biomedical and behavior-focused approaches that obscure the structural origins of disease. The case of SAWC-Boston illustrates the transformative potential of community-led, trauma-informed public mental health models that center dignity, cultural responsiveness, and trust-building. Such approaches not only improve engagement with healthcare and research but also address the root conditions that shape long-term cardiovascular risk.

Advancing cardiovascular health equity for South Asian communities therefore requires a paradigm shift toward integrated public mental health strategies that address trauma, disaggregate data, and invest in community-rooted interventions. Centering lived experience and collective agency is essential for reducing chronic disease burden and promoting health justice within historically marginalized populations.

## Data Availability

No new data were created or analyzed in this study. Data sharing is not applicable to this article as it is a theoretical analysis based on existing literature and community-based observations.

## Abbreviations

The following abbreviations are used in this manuscript:

CVD	Cardiovascular disease
SAWC	South Asian Workers’ Center
SAMSHA	Substance Abuse and Mental Health Services Administration
SDOH	Social Determinants of Health (Contextual)
NIH	National Institute of Health
SES	Socioeconomic Status (Contextual)
